# On the use of the coefficient of variation to quantify and compare trait variation

**DOI:** 10.1002/evl3.171

**Published:** 2020-05-14

**Authors:** Christophe Pélabon, Christoffer H. Hilde, Sigurd Einum, Marlène Gamelon

**Affiliations:** ^1^ Centre for Biodiversity Dynamics, Department of Biology Norwegian University of Science and Technology (NTNU) Trondheim 7491 Norway

**Keywords:** Dimensional analyses, genetic variance, measurement theory, quantitative genetics, teaching

## Abstract

Meaningful comparison of variation in quantitative trait requires controlling for both the dimension of the varying entity and the dimension of the factor generating variation. Although the coefficient of variation (CV; standard deviation divided by the mean) is often used to measure and compare variation of quantitative traits, it only accounts for the dimension of the former, and its use for comparing variation may sometimes be inappropriate. Here, we discuss the use of the CV to compare measures of evolvability and phenotypic plasticity, two variational properties of quantitative traits. Using a dimensional analysis, we show that contrary to evolvability, phenotypic plasticity cannot be meaningfully compared across traits and environments by mean‐scaling trait variation. We further emphasize the need of remaining cognizant of the dimensions of the traits and the relationship between mean and standard deviation when comparing CVs, even when the scales on which traits are expressed allow meaningful calculation of the CV.

**Impact Summary**

Statistical analyses in ecology and evolution often involve the calculation of summary statistics to facilitate interpretation. However, the transformation of the data involved in these calculations are often performed with little attention given to the meaning of the numbers. In some cases, this compromises the meaning of the analyses and undermines the conclusions of the studies. We illustrate this problem by showing how the calculation of the coefficient of variation (CV), a mean‐standardized measure of variation regularly used to quantify and compare variation of phenotypic traits, can become meaningless if one does not pay attention to the dimension of the entities measured, the scale on which these entities are measured and the relationship between the mean and the measure of variation. To minimize these common mistakes, we advocate a stronger emphasis on the meaning of the numbers when teaching quantitative methods.

Advanced statistical models to handle increasingly large and complex datasets are often employed at the expense of attention given to the meaning of the numbers (Houle et al. 2011; Tarka et al. 2015). This issue affects several aspects of the scientific process, from the measurement procedures to the interpretation of the statistical analyses where biological significance is often confounded with statistical significance (Yoccoz 1991; Tarka et al. 2015; Wasserstein and Lazar 2016). Here, we show that even the use of simple statistics such as the coefficient of variation (CV; standard deviation divided by the mean) can become uninformative or worse if attention is not paid to the meaning of the numbers when the CV is used to compare variation among quantitative traits.

Phenotypic plasticity and evolvability are two aspects of the variation of quantitative traits. Phenotypic plasticity corresponds to the variation expressed by a genotype when exposed to different environments (Bradshaw 1965; Schlichting 1986; DeWitt and Scheiner 2004), and evolvability (sensu Houle 1992) is the ability of a trait to respond to selection. Various measurements have been developed to quantify phenotypic variation produced by a given change in the environment or a given strength of selection. These have shown that quantitative traits differ in their sensitivity to environmental variation and in their ability to respond to selection, suggesting that both phenotypic plasticity and evolvability vary across traits, populations, and species (Mousseau and Roff 1987; Falconer 1989; Houle 1992; DeWitt and Scheiner 2004; Valladares et al. 2006, 2014). To unravel the causes of such variation and predict the ability of organisms to adapt, many studies have compared phenotypic plasticity and evolvability across traits, organisms, and populations using different methods for standardizing variation (e.g., Daehler 2003; Davidson et al. 2011; Palacio‐López and Gianoli 2011; Matesanz and Ramírez‐Valiente 2019, for phenotypic plasticity, and Mousseau and Roff 1987; Houle 1992; Merilä and Sheldon 2000; Hansen et al. 2011, for evolvability). Recently, the CV or related statistics expressing variation in relation to the mean (e.g., CV^2^) has been used to measure and compare both types of variation across traits (Fajardo and Piper 2011; Roscher et al. 2018; Acasuso‐Rivero et al. 2019, for phenotypic plasticity, and Houle 1992; Hansen et al. 2003, 2011, for evolvability).

Here, we show that despite apparent similarities, evolvability and phenotypic plasticity have different properties that prevent the use of CVs for comparing phenotypic plasticity across traits and environments. We then reiterate the cautions already expressed by several authors about the constraints imposed by the calculation of CVs on the scale of the measurement and on the mean‐standard deviation relationship, and we show how ignoring these caveats when comparing trait variation may jeopardize the interpretation and the conclusions of such comparisons.

## Measuring Evolvability and Phenotypic Plasticity

**Figure 1:**
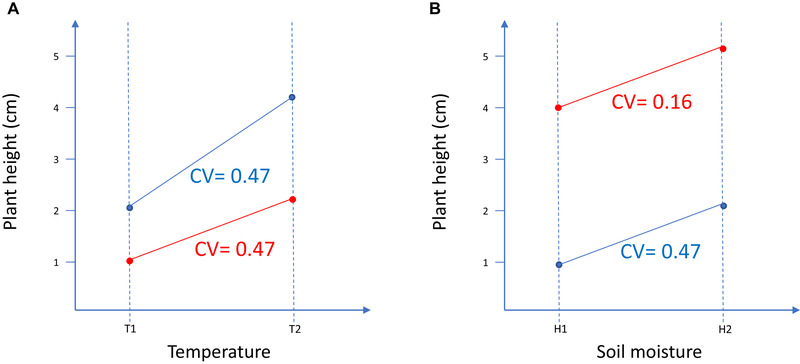
Reaction norms for one trait, plant height, measured for two genotypes (red and blue) in two different environmental gradients, temperature on the left and soil moisture on the right. In the two experiments, plasticity is measured for each genotype as the difference in phenotypic value divided by the change in either temperature or moisture. Thus, on the left phenotypic plasticity is expressed as cm °C^−1^, whereas on the right it is expressed as cm% humidity^−1^. To compare this variation, one can calculate the CV of the traits for each genotype in each experiment (CVs are reported with the color of the corresponding genotype). The CV of the two genotypes can be meaningfully compared within each experiment because the range of environmental variation over which CVs are estimated is similar. However, any comparison of CVs among experiments (i.e., among environmental gradients) is meaningless because °C cannot be compared with % humidity.

Following Houle (1992), evolvability can be estimated as the phenotypic change resulting from a given strength of selection, that is, the ratio between the phenotypic change and the selection gradient: e=Δz/β. Phenotypic plasticity, on the other hand, is described by the reaction norm of a trait, that is, the relationship between the phenotype and the environment. Measures of phenotypic plasticity are generally derived from the reaction norm, and in the simplest case (i.e., linear relationship between the environment and the phenotype) phenotypic plasticity can be measured as the average change in the phenotype per change in the environment $\delta \;=\;\frac{z_{2}-z_{1}}{m_{2}-m_{1}}$, where *z*_1_ and *z*_2_ are the phenotypic mean values of the trait measured in the environments *m*_1_ and *m*_2_ (Morrissey and Liefting 2016; Fig. 1). Thus, both evolvability and phenotypic plasticity measure phenotypic changes in relation to their respective triggering factors, namely, selection and environmental variation.

Despite apparent similarities, these two measures have different properties that constrain their use for further comparison. A dimensional analysis of these two quantities illustrates this point (See Schneider 2009, Chapter 6, for an introduction to dimensional analysis). The dimension of evolvability corresponds to the dimension of the trait *z* divided by the dimension of the selection gradient β,

e=Δz/β

$$\;\left[e\right]=\left[z\right]\;{\left[\beta \right]}^{-1},$$

where the symbols between brackets indicate the dimensions of the parameters. Because the selection gradient is the slope of the regression of the relative fitness *w* on the trait *z*, the dimension of *e* is

e=Δz×σz2Δz×σz2covw,zcovw,z,

$$\;\left[e\right]=\left[z\right]{\left[z\right]}^{2}\;{\left[w\right]}^{-1}{\left[z\right]}^{-1},$$

that is,

$$\;\left[e\right]={\left[z\right]}^{2}\;{\left[w\right]}^{-1}.$$

Thus, evolvability has the dimension of the trait *z* squared divided by the dimension of relative fitness, *w*. Because relative fitness *w* is defined as the fitness divided by the mean fitness, it is a dimensionless number, and evolvability simply has the dimension of the trait squared, [*z*]^2^. This agrees with the Lande equation (Lande 1979), $\Delta z\;=V_{a}\;\beta$, where evolvability defined as the ratio between the response to selection Δz and the selection gradient β equals $V_{a}$, the additive genetic variance that has a dimension of the trait squared.

For phenotypic plasticity, a similar dimensional analysis shows that *δ* has the dimension of the trait *z* divided by the dimension of the environmental variable *m*:

δ=Δz/Δm,

$$\;\left[\delta \right]=\left[z\right]\;{\left[m\right]}^{-1}.$$

Thus, the dimension of phenotypic plasticity is more complex than the dimension of evolvability because it depends on both the dimension of the trait and the dimension of the environmental gradient across which phenotypic plasticity is measured (Forsman 2015).

## Using Mean‐Standardization to Compare Evolvability or Phenotypic Plasticity

To compare variation among traits with different means and dimensions, one can express variation proportionally to the traits’ mean by dividing the measure of variation by the trait mean. This is the case when calculating CVs or squared coefficients of variation (CV2=σ(z)2/σ(z)2z¯2z¯2; see Pélabon et al. 2011 for a discussion of the advantage of CV^2^). Dividing the standard deviation that has the same dimension as the trait by the trait mean provides a dimensionless number that expresses variation as a proportion of the mean, or as a percentage of the mean when multiplied by 100.

CV≡σzσzz¯z¯,

$$\;\left[\mathrm{CV}\right]=\left[z\right]\;\;{\left[z\right]}^{-1}={\left[z\right]}^{0}.$$

Houle (1992) suggested that evolvability can be expressed proportionally to the trait mean if measured as the coefficient of additive genetic variation $CV_{a}=\sigma_{a}/\bar{z}$, where $\sigma_{a}$ is the square root of $V_{a}$, the additive genetic variance. Hansen et al. (2003, 2011) further showed that measuring evolvability as the squared coefficient of genetic variance $\;(I_{A}=V_{a}/{\bar{z}}^{2})$ facilitates interpretation by making evolvability a proportional change in trait mean when the trait experiences a selection gradient of 1, that is, a selection as strong as selection on fitness itself. Using CV_*a*_ or *I_A_* to compare evolvability of different traits is valid because it provides a dimensionless number comparable across traits. Considering *I_A_*,

$$I_{A}\equiv \;e/{\bar{z}}^{2}=V_{a}/{\bar{z}}^{2},$$

$$\;\left[I_{A}\right]={\left[z\right]}^{2}\;\;{\left[z\right]}^{-2}={\left[z\right]}^{0}.$$

Notice that *I_A_* represents an elasticity, that is, a proportional change in the trait per proportional change in fitness (van Tienderen 2000; Caswell 2001; Hansen et al. 2003, 2011).

In contrast, dividing plasticity *δ* by the trait mean does not provide a dimensionless measure of variation equivalent to a CV:

$$\;\delta /\bar{z}=\;\frac{\Delta_{z}/\Delta_{m}}{\bar{z}},$$

$$\left[\delta \right]\;{\left[z\right]}^{-1}=\left[z\right]\;{\left[m\right]}^{-1}\;{\left[z\right]}^{-1}={\left[m\right]}^{-1}.$$

Thus, dividing a measure of plasticity by the trait mean provides a measure of trait variation proportional to the trait mean per unit change of the environmental factor. Because this quantity is not dimensionless, it cannot be compared meaningfully when plasticity is measured across different environmental gradients (Fig. 1).

Several studies comparing phenotypic plasticity have acknowledged this issue. For example, studies comparing phenotypic plasticity between native and invasive species have used pairwise comparisons of the CV only when plasticity of the native and invasive species was measured on the same traits across identical environmental gradients, thus avoiding comparing variation generated by different environmental factors (within experiment comparison in Fig. 1; Daehler 2003; Davidson et al. 2011; Palacio‐López and Gianoli 2011). In contrast, comparing mean standardized phenotypic plasticity of traits measured along different environmental gradients (among experiment comparison in Fig. 1; e.g., Murren et al. 2014; Acasuso‐Rivero et al. 2019) is meaningless.

In theory, mean‐standardization of both trait variation and environmental variation would allow expressing phenotypic plasticity as an elasticity (i.e., a proportional change in the trait for a proportional change of the environmental factor), thus offering the possibility of comparing phenotypic plasticity across traits and environments. Such an approach was used by Wellstein et al. (2013) to test the relationship between intraspecific variation in plant traits and the variation of environmental parameters such as light, soil moisture, temperature, pH, and soil nutrients. Unfortunately, environmental gradients along which phenotypic plasticity is often estimated (e.g., temperature, latitude, presence‐absence of predators, and food availability) are often expressed on ordinal, nominal, or interval scales that do not allow meaningful calculation of the CV (Box 1). Because CVs are meaningful only for variables expressed on ratio or log‐interval scale (Lewontin 1966; Yablokov 1974; Hansen et al. 2011; Houle et al. 2011), the use of elasticity to compare phenotypic plasticity among traits and environments is most likely restricted to very specific cases.

Alternatively, one could divide the change in the environmental variable by its standard deviation. Combined with the mean‐standardization of the change in the trait, this provides a measure of phenotypic plasticity where a proportional change in the trait is generated by a change in environmental factor of one standard deviation. Assuming that the variation of the different environmental factors has been measured in the natural environment, and that this variation is symmetrically distributed around the mean, such a measure of plasticity would allow meaningful comparison of the phenotypic variation among traits and among environments, based on the relative variation of the environmental factors. However, comparing such measures would be meaningless for phenotypic variation estimated in experiments where the magnitude of the variation of the environmental factor is fixed by the experimental design and generally chosen to generate detectable changes in the phenotypic traits.

## Further Caveats While Using CVs to Compare Trait Variation

The CV expresses variation of an entity on a proportional scale that is easily interpretable when comparing variation among entities. If this remains the only goal for computing CVs, the only restriction for this computation concerns the scale on which entities are measured (Table 1, Box 1). However, interpreting differences among CVs may be seriously compromised unless the mathematical properties of the CV and the constraints imposed by the calculation of the CV on the properties of the trait distribution are considered (e.g., Lewontin 1966; Yablokov 1974; Lande 1977; Houle 1992; Gingerich 1993; Garcia‐Gonzalez et al. 2012).

For many traits (e.g., mass, metabolic rate, and length measurements), the standard deviation increases with the mean, and it is often assumed that the CV provides a measure of variation independent of the mean. This is true, however, only when the increase in the standard deviation is proportional to the increase in the mean (i.e., power of 2 in the Taylor power law between the variance and the mean; *σ* ^2^ = *aμ* ^2^, Taylor 1961). Unfortunately, as noticed by Van Valen (2005) about this proportionality: “*This is so often true that we may tend to forget that there are cases where it is not*.” For example, nonproportionality between the standard deviation and the mean is revealed by the negative relationship often observed between CV and trait mean of linear measurements of morphological traits (Bader and Hall 1960; Yablokov 1974; Soulé 1982; Pengilly 1984) or by the positive relationship observed between the CV and the mean body mass in mammals and birds (Hallgrímsson and Maiorana, 2000). Although a negative relationship between traits mean and CV can result from the effect of size‐independent measurement error (and can therefore be accounted for; Lande 1977; Rohlf et al. 1983), other factors may generate such a nonproportionality (see below). Yet, in many studies, differences in CV have been interpreted as resulting from biological/ecological differences (e.g., differences in the fitness‐trait relationship or differences in the intensity of competition) without testing the proportionality between the standard deviation and the mean, that is, without testing whether the CV truly provides a mean‐independent measure of variation. As noticed by Einum et al. (2012), the problem is even deeper because we generally do not have a null hypothesis concerning the relationship between the mean and the standard deviation, that is, we do not know what such a relationship would be in absence of external (i.e., ecological) factors affecting variation.

The nonproportionality between the mean and the standard deviation is not problematic if one's goal is to quantify or predict variation. For example, if two traits have different evolvabilities (*I_A_*), it means that the trait with the highest evolvability will evolve proportionally more than the trait with the lowest evolvability when exposed to selection of similar strength, whether or not the mean and the standard deviation change proportionally among traits. However, further interpretation of such a difference in evolvability should consider the possibility that this difference results from a nonproportional relationship between the mean and the standard deviation. Understanding the causes for such nonproportionality may become critical for interpreting differences in variation among quantitative traits. Below, we present some of the most common causes for nonproportionality between the mean and the standard deviation and we discuss the consequences of these when comparing variation.

Lande (1977) showed that CVs of objects measured as length, area, or volume are expected to differ according to the number of dimensions of the measurement (length = 1, area = 2, and volume = 3) and the correlations between these dimensions. Thus, for objects of constant shape, that is, with a correlation of one between the different linear measurements (length, height, and width), the CV of a volume (i.e., length^3^) will be three times the CV of the length, whereas the CV of an area (length^2^) will be twice the CV of the length. Consequently, we expect mass measurements to have larger CVs than area or length measurements. If the objects vary in shape and size, these factors (2 and 3) are expected to be upper limits of the multiplicative difference in CVs between objects. Additionally, for complex traits composed of multiple parts that covary, the increase of the standard deviation with the mean depends on the sign of the covariance, a positive covariance (i.e., *coordinated variation*) leads to a steeper increase of the variation (Taylor power >2), whereas a negative covariance (*compensatory variation*) leads to a shallower increase (Taylor power < 2; Mitteroecker et al. 2020). When computing the phenotypic CV for length and mass measurements of the data gathered by Hansen et al. (2011), we found that the average CV for mass measurements was more than an order of magnitude larger than for length measurements (average ± SE of CV_mass_ = 3.15 ± 1.11, *n* = 38; CV_length_ = 0.16 ± 0.03, *n* = 203, SE obtained by nonparametric bootstrapping), thus suggesting that several factors such as dimensions, correlations among dimensions, and complexity of the traits can simultaneously affect the value of the CVs.

**Figure 2:**
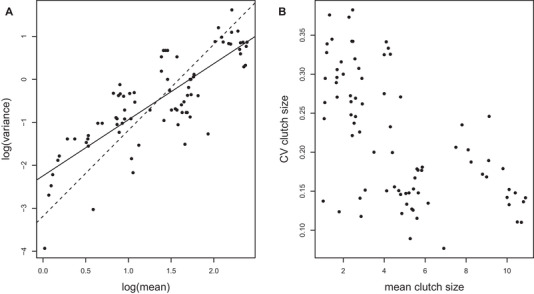
Variance‐mean relationship (A) and its effect on the CV (B) for phenotypic variation in clutch size in 32 bird species. The solid line in panel (A) represents the estimated increase in variance in clutch size with an increase in the mean (Taylor power, *b* = 1.31 ± 0.11). Because this slope is shallower than 2 (dash line), the standard deviation does not increase proportionally with the mean and the CV of clutch size decreases with an increasing mean clutch size (B; *r* = –0.58; 95% CI = –0.71, –0.42) (see Supporting Information for the data included in this analysis).

Nonproportionality between the mean and the standard deviation may also result from traits described by statistical distributions that differ from normal or log‐normal distributions. Indeed, for some distributions, we explicitly expect nonproportionality between the mean and the standard deviation. In Figure 2, we present such an example with the variation in clutch size among 32 bird species. Because clutch sizes in birds and litter sizes in mammals do not follow a normal or log‐normal distribution, the mean and the standard deviation are not expected to vary proportionally. Accordingly, the CV in clutch size decreases with an increasing mean clutch size (Fig. 2, the problem is the same for meristic traits). Therefore, if clutch sizes have on average lower CV in species with larger clutches compared to species with smaller clutches, one should be cautious when interpreting this difference. Of course, the nonproportionality between the mean and the standard deviation and the resulting difference in CVs between small and large clutches may reflect true biological differences in the variability of clutch size when expressed on a proportional scale (it may be easier to double a clutch of one egg than a clutch of six eggs), but further interpretation of the differences in the CVs should account for this effect before considering other factors such as the trait‐fitness relationship, or the effect of environmental variation. For traits expressed with binomial distributions such as probability to survive or reproduce, the specific relationship between the mean and the variance (maximum variance for *P* = 0.5 and zero for *P* = 0 or 1) generates CVs approaching infinity or zero for small and large values of *P*, respectively. Although a standardization of such CVs has been suggested (i.e., divided by the maximum possible CV; Morris and Doak 2004), these relativized CVs are not comparable to CVs estimated for traits with normal or log‐normal distribution (Hilde Christoffer et al. 2020).

**Box 1: Scale types, permissible transformations, and meaningful CV**

When performing measurements, we assign numbers to entities so that the relationship among numbers reflects an empirical relationship of interest among entities. Scales are imposed by these empirical relationships and the different types of scale are defined by the possible transformations of the numbers that preserve the empirical relationship, so called, permissible transformations (Stevens 1968; Hand 2004, Houle et al. 2011). For example, in an interval scale, permissible transformation should preserve the order of the numbers and the interval between two numbers and thus, only a monotonically increasing function is permissible. In the ratio scale, permissible transformation should preserve the order of the number as well as the order of the differences and ratios between entities. On this scale, only multiplication by a constant is permissible. For example, if four individuals have mass of *a* = 5, *b* = 10, *c* = 20, and *d* = 22 g, respectively, multiplying each number by 2 preserves the order of the difference (*b* – *a* > *d* – *c* and 2*b* – 2*a* > 2*d* – 2*c*), as well as the order of the ratio (*b*/*a* > *d*/*c* and 2*b*/2*a* > 2*d*/2*c*). However, raising the numbers to a power of 2 does not preserve the magnitude of the differences (*b* – *a* > *d* – *c* but *b* ^2^ – *a* ^2^ < *d* ^2^ – *c* ^2^). In the log‐interval scale, only the order between the ratios should be preserved by transformation, and the power transformation becomes permissible (*b*/*a* > *d*/*c* and *b* ^2^/*a* ^2^ > *d* ^2^/*c* ^2^). It is sometimes possible and meaningful to convert interval scale measurements into ratio scale measurements. For example, converting birth year to years since birth (i.e., age) allows meaningful comparison of the new values by taking their ratio (I am now three times older than my daughter), whereas the ratio of our birth years is meaningless.

Meaningful CVs can only be calculated for a restricted number of scales. For any scales where the zero point is not defined (nominal scale and ordinal scale) or arbitrarily chosen (interval scale), it is not meaningful to calculate a CV and talk about proportional changes. Similarly, the calculation of the CV may be compromised for any scale where the mean can be equal to 0 (signed‐ratio scale or difference scale; in the difference scale, the zero point corresponds to ln(1)). Notice that a clearly defined zero point does not necessarily mean that 0 has a clear biological meaning. For example, if we use gram or centimeter to measure the size of some individual organisms, these two measurements have a clearly defined 0, but we do not expect to observe individuals of 0 g or 0 cm. Finally, for absolute scales such as probability, the calculation and the interpretation of the CV may be strongly affected by the distribution of the data and the mean‐standard deviation relationship (see main text). Table 1 summarizes the different scales, their permissible transformation, and whether the calculation of CVs is meaningful.

Still, in most studies that have used CVs to compare variation among traits, authors have explained the observed differences without testing the proportionality between the mean and the standard deviation and without accounting for the possible effect of among‐trait differences in dimensionality on the value of the CV (e.g., Blanck and Lamouroux 2007; Greenway and Harder 2007; Fajardo and Piper 2011; Roscher et al. 2018; Acasuso‐Rivero et al. 2019). At best, such practice introduces variation in the CVs that decreases the statistical power of any comparisons between groups of traits (e.g., Acasuso‐Rivero et al. 2019). In other cases, this may lead to potentially erroneous conclusions. For example, in the study by Roscher et al. (2018), differences in CVs between traits related to gas exchange and growth on the one hand and traits related with leaf morphology, anatomy, and photochemistry on the other hand were interpreted as due to differences in the “level of organization,” whereas the two groups of traits markedly differed in their dimensions and statistical distribution (see Ramírez‐Valiente et al. 2018 for a similar issue). Although the conclusions of these studies may turn out to be correct, they remain questionable as long as the possible effects of a nonproportional increase of the standard deviation with the mean on the CVs have not been considered.

## Conclusions

The problems exposed here are common in the literature in ecology and evolution where using the CV as a dimensionless measure of variation is widespread. Many studies have calculated CVs from variables on signed‐ratio scale (i.e., variables taking both positive and negative values; e.g., stigmatic exertion, Larrinaga et al. 2009; style deflexion, Dai et al. 2016) or interval scale (temperature, Sammarco et al. 2006; reflectance spectrum, chroma, or hue, Mennill et al. 2003; Ibáñez et al. 2013; Jacobs et al. 2015; Charmantier et al. 2017), and many studies have compared CVs between traits with different dimensions or statistical distribution. Notice that variance‐standardization (e.g., *Z*‐transformation, heritability, and selection intensity) is often subject to similar shortcoming when it comes to compare variation (Hereford et al. 2004; Hansen et al. 2011; Houle et al. 2011; Matsumura et al. 2012). More generally, standardization and transformation of data are routinely performed before data analyses without paying attention to the consequences of these manipulations on the meaning of the numbers. In many cases, this renders the conclusions of the studies questionable. We believe that dimensional analyses as the one performed here (see also Schneider 2009) and a better awareness of the different types of measurement scale should become standard tools (i.e., taught along with statistical analyses in quantitative biology classes) to assess the meaning and the validity of the statistics or summary statistics used in ecology and evolution.

**Table 1:** Scale types, permissible transformations, and meaningful calculation of CV

Scale type	Domain	Permissible transformation(s)	Biological examples	Meaningful CV
Nominal	Any set of symbols	Any one‐to‐one substitution	Species, genes, color (when described as name), number assigned to football players	No
Ordinal	Ordered symbols	Any monotonically increasing function	Dominance, birth order	No
Interval	Real number	Linear transformation k(x)=b+ax	Dates, latitude, pH, color in the RGB domain, reflectance spectrum	No
Log‐interval	Positive real number	$k\;\left(x\right)=\gamma_{k}\;x^{\delta_{k}}$, where $\gamma_{k}$ and $\delta_{k}\;>0$	Body size	Yes
Difference	Real numbers	Addition of a constant k(x)=a+x	Log‐transformed variables initially on a ratio scale	No^1^
Ratio	Positive real number	Multiplication by a constant k(x)=ax	Mass, length, duration, specific leaf area	Yes
Signed ratio	Real numbers	Multiplication by a constant k(x)=ax	Intrinsic growth rate, signed asymmetry, stigmatic exertion, residuals from linear models^2^	Yes / No^3^
Absolute	Defined	None	Probability	Yes/No^4^

1 The log‐transformation of the data changes the meaning of the zero point and the calculation of the CV loses its meaning. Furthermore, if the variable has a mean of 1 on the original scale, it will have a mean of 0 in the difference scale and this will prevent the calculation of the CV.

2 Calculating the CV of the residuals of a linear model can be done by using the average value of the response variable as trait mean.

3 Calculating the CV is allowed if all the numbers in the distribution have the same sign (notice that this could generate negative CVs). However, cautions should be taken when calculating and interpreting CVs when the distribution comprises both positive and negative numbers. Because the zero point has a clear definition in this case, both mathematically and biologically, the CV may be meaningful, but its value may be extreme or even undefined (i.e., +∞) when the mean is close or equal to 0 (e.g., see Pélabon and Hansen 2008).

4 For probabilities, the variance has a value of zero for *P* = 0 and *P* = 1, and the CV varies between +∞ and 0 when the value of the probability varies from 0 to 1. Morris and Doak (2004) suggested to calculate a relativized CV defined as the observed CV divided by the maximum CV, that is, the CV obtained with the maximal variance possible for a given average probability. Because this relativized CV corresponds to a proportion of the CV, it cannot be compared with CVs calculated for traits that are on other scale types.

## CONFLICT OF INTEREST

The authors declare no conflict of interest.

Associate Editor: A. Charmantier

## Supporting information

Supplementary Material

## References

[evl3171-bib-0001] Acasuso‐Rivero, C. , C. J. Murren , C. D. Schlichting , and U. K. Steiner . 2019 Adaptive phenotypic plasticity for life‐history and less fitness‐related traits. Proc. R. Soc. B 286:20190653. 10.1098/rspb.2019.0653 PMC6571476 31185861

[evl3171-bib-0002] Bader, R. S. , and J. S. Hall . 1960 Osteometric variation and function in bats. Evolution 14:8–17.

[evl3171-bib-0003] Blanck, A. , and N. Lamouroux . 2007 Large‐scale intraspecific variation in life‐history traits of European freshwater fish. J. Biogeogr. 34:862–875.

[evl3171-bib-0004] Bradshaw, A. D. 1965 Evolutionary significance of phenotypic plasticity in plants. Adv. Genet. 13:115–55.

[evl3171-bib-0005] Caswell, H. 2001 Matrix population models: construction, analysis, and interpretation. 2nd ed. Sinauer, Sunderland, MA.

[evl3171-bib-0006] Charmantier, A. , M. Wolak , A. Grégoire , A. Fargevieille , and C. Doutrelant . 2017 Colour ornamentation in the blue tit: quantitative genetic (co) variances across sexes. Heredity 118:125–134. 27577691 10.1038/hdy.2016.70 PMC5234477

[evl3171-bib-0007] Daehler, C. C. 2003 Performance comparisons of co‐occurring native and alien invasive plants: implications for conservation and restoration. Ann. Rev. Ecol. Syst. 34:183–211.

[evl3171-bib-0008] Dai, C. , X. Liang , J. Ren , M. Liao , J. Li , and L. F. Galloway . 2016 The mean and variability of a floral trait have opposing effects on fitness traits. Ann. Bot. 117:421–429. 26749589 10.1093/aob/mcv189 PMC4765544

[evl3171-bib-0009] Davidson, A. M. , M. Jennions , and A. B. Nicotra . 2011 Do invasive species show higher phenotypic plasticity than native species and, if so, is it adaptive? A meta‐analysis. Ecol. Lett. 14:419–431. 21314880 10.1111/j.1461-0248.2011.01596.x

[evl3171-bib-0010] DeWitt, T. J. , and S. M. Scheiner . 2004 Phenotypic plasticity: functional and conceptual approaches. Oxford Univ. Press, Oxford, U.K.

[evl3171-bib-0011] Einum, S. , T. Forseth , and A. G. Finstad . 2012 Individual variation in response to intraspecific competition: problems with inference from growth variation measures. Methods Ecol. Evol. 3:438–444.

[evl3171-bib-0012] Fajardo, A. , and F. I. Piper . 2011 Intraspecific trait variation and covariation in a widespread tree species (*Nothofagus pumilio*) in southern Chile. New Phytol. 189:259–271. 21039558 10.1111/j.1469-8137.2010.03468.x

[evl3171-bib-0013] Falconer, D. S. 1989 Introduction to quantitative genetics. 3rd ed. Longman Scientific & Technical, Harlow, U.K.

[evl3171-bib-0014] Forsman, A. 2015 Rethinking phenotypic plasticity and its consequences for individuals, populations and species. Heredity 115:276–285. 25293873 10.1038/hdy.2014.92 PMC4815454

[evl3171-bib-0015] Garcia‐Gonzalez, F. , L. W. Simmons , J. L. Tomkins , J. S. Kotiaho , and J. P. Evans . 2012 Comparing evolvabilities: common errors surrounding the calculation and use of coefficients of additive genetic variation. Evolution 66:2341–2349. 22834736 10.1111/j.1558-5646.2011.01565.x

[evl3171-bib-0016] Gingerich, P. D. 1993 Quantification and comparison of evolutionary rates. Am. J. Sci. 293:453–478.

[evl3171-bib-0017] Greenway, C. A. , and L. D. Harder . 2007 Variation in ovule and seed size and associated size–number trade‐offs in angiosperms. Am. J. Bot. 94:840–846. 21636453 10.3732/ajb.94.5.840

[evl3171-bib-0018] Hallgrímsson, B. , and V. Maiorana . 2000 Variability and size in mammals and birds. Biol. J. Linn. Soc. 70:571–95.

[evl3171-bib-0019] Hand, D. J. 2004 Measurement theory and practice: the world through quantification. Arnold, Lond.

[evl3171-bib-0020] Hansen, T. F. , C. Pélabon , W. S. Armbruster , and M. L. Carlson . 2003 Evolvability and genetic constraint in *Dalechampia* blossoms: components of variance and measures of evolvability. J. Evol. Biol. 16:754–66. 14632238 10.1046/j.1420-9101.2003.00556.x

[evl3171-bib-0021] Hansen, T. F. , C. Pélabon , and D. Houle . 2011 Heritability is not evolvability. Evol. Biol. 38:258–277.

[evl3171-bib-0022] Hereford, J. , T. F. Hansen , and D. Houle . 2004 Comparing strengths of directional selection: how strong is strong? Evolution 58:2133–143. 15562680 10.1111/j.0014-3820.2004.tb01592.x

[evl3171-bib-0023] Hilde C. H. , M. Gamelon , B.‐E. Sæther , J.‐M. Gaillard , N. G. Yoccoz , and C. Pélabon . 2020 The demographic buffering hypothesis: evidence and challenges. Trends in Ecology & Evolution. 10.1016/j.tree.2020.02.004. 32396819

[evl3171-bib-0024] Houle, D. 1992 Comparing evolvability and variability of quantitative traits. Genetics 130:195–204. 1732160 10.1093/genetics/130.1.195 PMC1204793

[evl3171-bib-0025] Houle, D. , C. Pélabon , G. P. Wagner , and T. F. Hansen . 2011 Measurement and meaning in biology. Q. Rev. Biol. 86:3–34. 21495498 10.1086/658408

[evl3171-bib-0026] Ibáñez, A. , A. Marzal , P. López , and J. Martín . 2013 Sexually dichromatic coloration reflects size and immunocompetence in female Spanish terrapins, *Mauremys leprosa* . Naturwissenschaften 100:1137–47. 24253419 10.1007/s00114-013-1118-2

[evl3171-bib-0027] Jacobs, A. C. , J. M. Fair , and M. Zuk . 2015 Coloration, paternity, and assortative mating in western bluebirds. Ethology 121:176–186.

[evl3171-bib-0028] Lande, R. 1977 Comparing coefficients of variation. Syst. Zool. 26:214–217.

[evl3171-bib-0029] Lande, R. 1979 Quantitative genetic analysis of multivariate evolution applied to brain‐body size allometry. Evolution 33:402–416. 28568194 10.1111/j.1558-5646.1979.tb04694.x

[evl3171-bib-0030] Larrinaga, A. R. , P. Guitián , J. L. Garrido , and J. Guitián . 2009 Floral morphology and reproductive success in herkogamous *Narcissus cyclamineus* (Amaryllidaceae). Plant Syst. Evol. 278:149–157.

[evl3171-bib-0031] Lewontin, R. C. 1966 On the measurement of relative variability. Syst. Zool. 15:141–142.

[evl3171-bib-0032] Matesanz, S. , and J. A. Ramírez‐Valiente . 2019 A review and meta‐analysis of intraspecific differences in phenotypic plasticity: implications to forecast plant responses to climate change. Global Ecol. Biogeogr. 28:1682–1694.

[evl3171-bib-0033] Matsumura, S. , R. Arlinghaus , and U. Dieckmann . 2012 Standardizing selection strengths to study selection in the wild: a critical comparison and suggestions for the future. Bioscience 62:1039–1054.

[evl3171-bib-0034] Mennill, D. J. , S. M. Doucet , R. Montgomerie , and L. M. Ratcliffe . 2003 Achromatic color variation in black‐capped chickadees, *Poecile atricapilla*: black and white signals of sex and rank. Behav. Ecol. Sociobiol. 53:350–357.

[evl3171-bib-0035] Merilä, J. , and B. Sheldon . 2000 Lifetime reproductive success and heritability in nature. Am. Nat. 155:301–310. 10718727 10.1086/303330

[evl3171-bib-0036] Mitteroecker, P. , S. Bartsch , C. Erkinger , N. D. S. Grunstra , A. Le Maître , and F. L. Bookstein . 2020 Morphometric variation at different spatial scales: coordination and compensation in the emergence of organismal form. Evolution: *In press*. 10.1093/sysbio/syaa007 PMC7440742 32011716

[evl3171-bib-0037] Morris, W. F. , and D. F. Doak . 2004 Buffering of life histories against environmental stochasticity: accounting for a spurious correlation between the variabilities of vital rates and their contributions to fitness. Am. Nat. 163:579–590. 15122504 10.1086/382550

[evl3171-bib-0038] Morrissey, M. B. , and M. Liefting . 2016 Variation in reaction norms: statistical considerations and biological interpretation. Evolution 70:1944–1959. 27431762 10.1111/evo.13003

[evl3171-bib-0039] Mousseau, T. A. , and D. A. Roff . 1987 Natural‐selection and the heritability of fitness components. Heredity 59:181–97. 3316130 10.1038/hdy.1987.113

[evl3171-bib-0040] Murren, C. J. , H. J. Maclean , S. E. Diamond , U. K. Steiner , M. A. Heskel , C. A. Handelsman , etal. 2014 Evolutionary change in continuous reaction norms. Am. Nat. 183:453–467. 24642491 10.1086/675302

[evl3171-bib-0041] Palacio‐López, K. , and E. Gianoli . 2011 Invasive plants do not display greater phenotypic plasticity than their native or non‐invasive counterparts: a meta‐analysis. Oikos 120:1393–1401.

[evl3171-bib-0042] Pélabon, C. , and T. F. Hansen . 2008 On the adaptive accuracy of directional asymmetry in insect wing size. Evolution 62:2855–2867. 18752614 10.1111/j.1558-5646.2008.00495.x

[evl3171-bib-0043] Pélabon, C. , W. S. Armbruster , and T. F. Hansen . 2011 Experimental evidence for the Berg hypothesis: vegetative traits are more sensitive than pollination traits to environmental variation. Funct. Ecol. 25:247–257.

[evl3171-bib-0044] Pengilly, D. 1984 Developmental versus functional explanations for patterns of variability and correlation in the dentitions of foxes. J. Mammal. 65:34–43.

[evl3171-bib-0045] Ramírez‐Valiente, J. A. , J. R. Etterson , N. J. Deacon , and J. Cavender‐Bares . 2018 Evolutionary potential varies across populations and traits in the neotropical oak *Quercus oleoides* . Tree Physiol. 39:427–439. 10.1093/treephys/tpy108 30321394

[evl3171-bib-0046] Rohlf, F. J. , A. J. Gilmartin , and G. Hart . 1983 The Kluge‐Kerfoot phenomenon‐a statistical artifact. Evolution 37:180–202. 28568033 10.1111/j.1558-5646.1983.tb05526.x

[evl3171-bib-0047] Roscher, C. , J. Schumacher , A. Lipowsky , M. Gubsch , A. Weigelt , B. Schmid , etal. 2018 Functional groups differ in trait means, but not in trait plasticity to species richness in local grassland communities. Ecology 99:2295–2307. 29989166 10.1002/ecy.2447

[evl3171-bib-0048] Sammarco, P. W. , A. Winter , and J. C. Stewart . 2006 Coefficient of variation of sea surface temperature (SST) as an indicator of coral bleaching. Mar. Biol. 149:1337–1344.

[evl3171-bib-0049] Schlichting, C. D. 1986 The evolution of phenotypic plasticity in plants. Ann. Rev. Ecol. Syst. 17:667–693.

[evl3171-bib-0050] Schneider, D. C. 2009 Quantitative ecology. Academic press, Amsterdam, the Netherlands.

[evl3171-bib-0051] Soulé, M. E. 1982 Allomeric variation. 1. The theory and some consequences. Am. Nat. 120:751–64.

[evl3171-bib-0052] Stevens, S. S. 1968 Measurement, statistics and the schemapiric view. Science 161:849–856. 5667519 10.1126/science.161.3844.849

[evl3171-bib-0053] Tarka, M. , G. H. Bolstad , S. Wacker , K. Rasanen , T. F. Hansen , and C. Pélabon . 2015 Did natural selection make the Dutch taller? A cautionary note on the importance of quantification in understanding evolution. Evolution 69:3204–3206. 26507881 10.1111/evo.12803

[evl3171-bib-0054] Taylor, L. R. 1961 Aggregation, variance and the mean. Nature 189:732–735.

[evl3171-bib-0055] Valladares, F. , D. Sanchez‐Gomez , and M. A. Zavala . 2006 Quantitative estimation of phenotypic plasticity: bridging the gap between the evolutionary concept and its ecological applications. J. Ecol. 94:1103–1116.

[evl3171-bib-0056] Valladares, F. , S. Matesanz , F. Guilhaumon , M. B. Araújo , L. Balaguer , M. Benito‐Garzón , etal. 2014 The effects of phenotypic plasticity and local adaptation on forecasts of species range shifts under climate change. Ecol. Lett. 17:1351–1364. 25205436 10.1111/ele.12348

[evl3171-bib-0057] van Tienderen, P. H. 2000 Elasticities and the link between demographic and evolutionary dynamics. Ecology 81:666–679.

[evl3171-bib-0058] Van Valen, L. 2005 The statistics of variation Pp. 29–47 *in* Hallgrimsson B. and Hall B. K., eds. Variation, a central concept in biology. Academic Press, Cambridge, MA.

[evl3171-bib-0059] Wasserstein, R. L. , and N. A. Lazar . 2016 The ASA statement on p‐values: context, process, and purpose. Am. Stat. 70:129–133.

[evl3171-bib-0060] Wellstein, C. , S. Chelli , G. Campetella , S. Bartha , M. Galiè , F. Spada , etal. 2013 Intraspecific phenotypic variability of plant functional traits in contrasting mountain grasslands habitats. Biodivers. Conserv. 22:2353–2374.

[evl3171-bib-0061] Yablokov, A. V. 1974 Variability of mammals. Amerind Publishing Co, New Delhi, India.

[evl3171-bib-0062] Yoccoz, N. G. 1991 Use, overuse, and misuse of significance tests in evolutionary biology and ecology. Bull. Ecol. S. Am. 72:106–11.

